# Tuneable 2D surface Bismuth incorporation on InAs nanosheets†

**DOI:** 10.1039/d3nr00454f

**Published:** 2023-05-12

**Authors:** Sandra Benter, Yi Liu, Renan Da Paixao Maciel, Chin Shen Ong, Lassi Linnala, Dong Pan, Austin Irish, Yen-Po Liu, Jianhua Zhao, Hongqi Xu, Olle Eriksson, Rainer Timm, Anders Mikkelsen

**Affiliations:** a NanoLund & Department of Physics, Lund University Box 118 22100 Lund Sweden anders.mikkelsen@sljus.lu.se; b Department of Physics and Astronomy, Materials Theory Box 516 751 20 Uppsala Sweden; c State Key Laboratory of Superlattices and Microstructures, Institute of Semiconductors, Chinese Academy of Sciences P.O. Box 912 Beijing 100083 China; d Center of Materials Science and Optoelectronics Engineering, University of Chinese Academy of Sciences Beijing 100190 China; e Beijing Key Laboratory of Quantum Devices, Key Laboratory for the Physics and Chemistry of Nanodevices and Department of Electronics, Peking University Beijing 100871 China; f Beijing Academy of Quantum Information Sciences Beijing 100193 China

## Abstract

The chemical bonding at the interface between compound semiconductors and metals is central in determining electronic and optical properties. In this study, new opportunities for controlling this are presented for nanostructures. We investigate Bi adsorption on 2D wurtzite InAs (112̄0) nanosheets and find that temperature-controlled Bi incorporation in either anionic- or cationic-like bonding is possible in the easily accesible range between room temperature and 400 °C. This separation could not be achieved for ordinary zinc blende InAs(110) surfaces. As the crystal structures of the two surfaces have identical nearest neighbour configurations, this indicates that overall geometric differences can significantly alter the adsorption and incorporation. *Ab initio* theoretical modelling confirms observed adsorption results, but indicate that both the formation energies as well as kinetic barriers contributes to the observed temperature dependent behaviour. Further, we find that the Bi adsorption rate can differ by at least 2.5 times between the two InAs surfaces while being negligible for standard Si substrates under similar deposition conditions. This, in combination with the observed interface control, provides an excellent opportunity for tuneable Bi integration on 2D InAs nanostructures on standard Si substrates.

## Abstract 2

First experimental study of 2D Bi incorporation into InAs WZ nanosheets in comparison to ZB bulk samples and DFT calculations.

## Graphical abstract

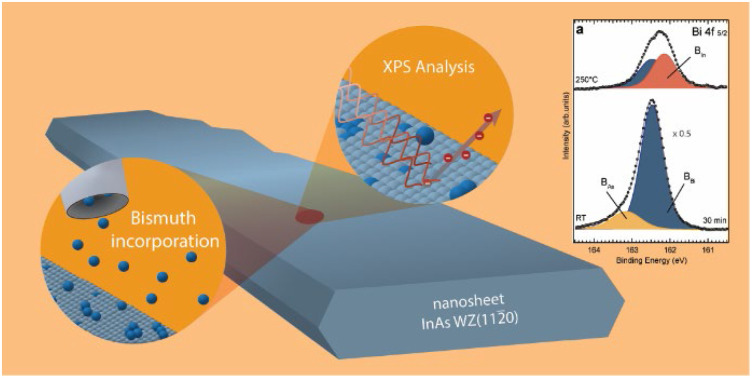

## Introduction

Central to the function of a wide range of devices, such as for quantum technologies^1^ and photovoltaics,^2,3^ is the atomic scale quality of the interface between a metal and the semiconductor. For covalently bound compound semiconductors, the interface can have both anionic and cationic bonding, which strongly influences interface properties. Fundamentally important examples are the formation of Schottky barriers^4,5^ and the introduction of a significant bandgap in 2D materials.^6,7^ Accordingly, interface bonding is one of the fundamental parameters that needs control to define the device performance.

The group V element bismuth is a peculiar material which acts both as a surfactant in III–V material growth^8^ and as a constituent in a number of quantum materials.^9^ From first principle calculations, it is known that if Bi is incorporated into the surface lattice of other III–V compounds band inversion^10^ with induced nontrivial topological properties,^11^ large spin–orbit coupling,^12^ as well as enhanced localization effects of charge carriers^13^ can be expected based on the formation of III-Bi alloys. InAs has gained in importance as one of the III–V semiconductor systems to outperform standard Si-based technologies. This compound material has great potential for infrared detectors,^14^ low-power electronics^15^ and quantum computing.^1^ InAs usually crystallises in a zinc blende (ZB) structure but can also be grown in a wurtzite (WZ) phase in low-dimensional structures. This opens the door to explore and create novel devices based on bandgap heterostructures^16,17^ along with a lower susceptibility to temperature and atmospheric conditions for subthreshold characteristics.^18^ Difficulties arise when trying to incorporate Bi into the InAs lattice. The large miscibility gap between regions of InBi and InAs^10^ resulting from the respective tetragonal and cubic lattice structure generates phase separation and clustering of Bi adatoms during the growth of bulk materials.^19^ Previous experimental studies focus on the formation of thin metallic Bi films on top of ZB bulk substrates.^20–23^ Bi is deposited for several monolayers on a sample at room temperature and subsequently annealed. As a result, reconstruction of the original InAs surface,^22,23^ additional components in the In and Bi core level spectra^21^ and a Rashba spin splitting exceeding 4–5 times that of other 1D/quasi Rashba systems^20^ were observed. Also, it was shown that creating a sharp Bi–semiconductor interface suppresses the formation of metal-induced gap states leading to very low contact resistance in the case of two-dimensional MoS_2_.^24^ These promising results pose the question of how to induce a stronger interaction and possible incorporation of Bi adatoms in the InAs surface lattice to induce the theoretically predicted characteristics for InAs:Bi compound systems while maintaining a high-quality interface to the pristine InAs.

**Fig. 1:**
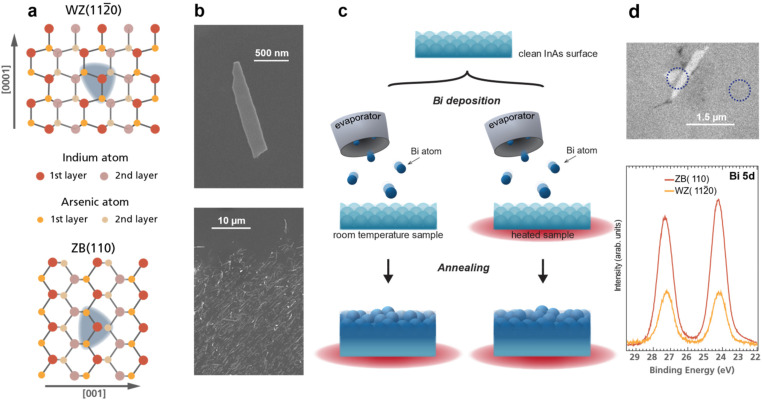
(a) Top view atomistic sketch of the InAs WZ(112̄0) and ZB(110) crystal facets. Both crystal structures exhibit the same nearest neighbour correlation (indicated by the blue area). Note the geometric differences arise for the second nearest neighbour. (b) Top: SEM image of a single InAs nanosheet after transferring it to a Si substrate. Bottom: SEM image displaying the density of deposited nanostructures for the XPS measurements in Fig. 2. (c) Schematic of the experimental procedure: after removing the native InAs oxide, Bi atoms are deposited on to the sample at room or elevated temperature followed by subsequent annealing steps. (d) Top: XPEEM image of InAs nanosheets indicating that spatially resolved measurements are achievable, allowing for clear distinction between signal from the nanostructures and the ZB substrate. Bottom: Bi 5d core level spectra from an InAs(110) substrate and a single InAs(112̄0) nanosheet after depositing Bi for 15 min while annealing the sample to 250 °C. Measurement positions are indicated by dashed lines in the XPEEM image above.

Here, we study Bi adsorption on the InAs WZ(112̄0) surface of nanosheets and compare it to the conventional ZB(110) bulk surface. These surface crystal structures are non-polar with an identical nearest neighbour configuration, see Fig. 1a. However, geometrical differences arise for the second nearest neighbour, thus giving a view into the importance of even subtle atomic-scale differences. X-ray photoemission spectroscopy (XPS) analysis is performed after each Bi deposition and annealing step on all sample types. Two thermal preparation procedures are explored, as depicted in Fig. 1c, to investigate the use of the sample temperature for deposition tuneability. We find that the adsorption rate, bonding, and incorporation mechanism strongly depends on the crystal facet. Further, only the WZ nanosheet surface allows significant tunability of the bonding configuration of the Bi atoms to InAs – switching between anionic and cationic type bonding. The high-resolution XPS measurements on nanostructure ensembles are complemented by measurements on individual nanosheets using X-ray photoemission electron microscopy (XPEEM) and scanning photoemisison microscopy (SPEM) that confirm the results and add additional information on the quantitative differences in adsorption. The experimental results are compared to density functional theory (DFT) calculations of the formation energies for Bi surface incorporation and vacancy formation. In combination, we conclude that while substitutional adsorption is favourable, kinetic barriers allow the formation of distinctly different phases on the nanosheets. Further, the adsorption rates differ significantly between both InAs surfaces as well as the Si substrate.

## Experimental and computational details

### InAs sample growth

InAs bulk and nanostructure samples were employed to investigate the incorporation mechanism of Bi into different crystal structures. N-type doped InAs(110) substrates were taken from commercially available wafers (*n* ∼ 10^18^ cm^−3^). The WZ(112̄0) crystal structures were grown in form of 20 nm thick nanosheets.^25^ The individual width ranged between 100–500 nm with an average length of 1.5 μm (example can be seen in Fig. 1b). Nanostructures were deposited onto Si or InAs(110) wafers covered with native oxide. The native In and As oxides on the InAs samples were removed *via* atomic hydrogen treatment. For this, the samples were heated to 400 °C and exposed to atomic hydrogen with a pressure of around 5 × 10^−6^ mbar. The annealing time ranged from 30 up to 60 min. A hydrogen atom beam source from MBE Komponenten was used, and the sample temperature was measured with a pyrometer. The oxide removal was validated using XPS measurements.

### Bi deposition

Bi was evaporated sequentially by heating a low-temperature effusion cell from MBE Komponenten to 400 ± 5 °C for 10 to 60 min. Three different sample temperature regions were explored: (i) deposition at room temperature, (ii) deposition at 290–300 °C as well as intermediate temperature ranges with (iii) deposition at 250–260 °C (discussed in the ESI†). Additionally, subsequent annealing steps were performed by ramping the sample fast and controlled to temperatures from 250 °C to 400 °C, and keeping the target value within ±10 °C for 10 min, respectively. The overall process is depicted in Fig. 1c. After each preparation and annealing step, XPS measurements were performed.

### XPS measurement and analysis

Synchrotron based XPS and microscopy experiments were carried out at the AU-Matline (Astrid, Aarhus-Denmark), ESCA Microscopy (Elettra, Trieste-Italy), MaxPEEM and FlexPES beamline (MaxIV, Lund-Sweden). All XPS measurements were performed at room temperature and under pressures <10^−9^ mbar. The high spatial resolution at the ESCA Microscopy and MaxPEEM beamline allowed for an investigation of individual nanostructures and bulk material for temperature regions (ii) and (iii). Furthermore, for temperature zone (i) and (ii) InAs(110) bulk substrates were measured with a larger beam spot size at Astrid, as well as sample areas with high densities of WZ(112̄0) crystal nanosheets on Si at FlexPES. For varying kinetic energies, Bi 4f (photon energy: 460 eV) and 5d (120 eV), In 3d (650 eV) and 4d (120 eV), as well as As 3d (140 eV and 650 eV) core level spectra were recorded for the regions of interest. The core levels for bulk and nanostructure samples were calibrated *via* Au 4f and Si 2p reference measurements, respectively. This excludes the measurements at MaxPEEM. Here, the photon energy was kept constant at 100 eV for all measurements allowing for a relative comparison of the acquired datasets. The XPS analysis of all core levels involved a background removal with a Shirley function. All datasets were fitted with Voigt functions for each component following the principle of including the minimum number of components necessary. The fits were carried out with a Lorentzian full width half maximum of 0.19 ± 0.03 eV, 0.18 ± 0.01 eV and 0.24 ± 0.02 eV for In, As and Bi core levels. In general, we determined three different components in the Bi 4f core level, one at 0.29 ± 0.04 eV lower binding energies and one at 0.56 ± 0.06 eV higher binding energies compared to the metallic Bi component (comparable to ref. 26). We attribute the additional components to Bi–In and Bi–As bonds, respectively. We estimate the thickness of deposited Bi films based on In and As core level spectra (detailed description in S7 in the ESI†).

Since different synchrotron setups were involved, the exact angle (target value 90°) and the distance between the sample and Bi source may differ which led to varying deposition times for individual preparation steps. This was considered in the interpretation of the data. We are confident that setup-induced fluctuations are not altering the observed effects as described here.

### DFT calculations

Density functional theory (DFT) calculations were performed using Quantum ESPRESSO^27^ for the InAs WZ(112̄0) and ZB(110) surface. The pristine WZ (ZB) surface has an armchair (zigzag) geometry that alternates the in-plane In and As atoms in the [0001]-([11̄0]-) direction. To model WZ(112̄0) as a slab, we constructed a two-dimensional (1 × 1) super-cell containing 44 atoms, with periodicity in the *a*- and *b*-directions with *a* = 7.24 Å, *b* = 7.49 Å and a thickness of approx. 20 Å in the out-of-plane direction. To model ZB(110), a two-dimensional (2 × 2) periodic slab super-cell containing 56 atoms, with lattice parameters of *a* = 12.11 Å, *b* = 8.56 Å, and a thickness of 11.45 Å in the out-of-plane direction was considered. To avoid interactions between the periodic images, a vacuum in the out-of-plane direction of 16 Å was included. Due to the broken symmetry in the out-of-plane direction resulting in the emergence of dangling bonds, we saturated the As and In atoms at the bottom surfaces of both slabs with pseudo-hydrogen with a nuclear charge of 0.75*e* and 1.25*e* respectively (where *e* is the positive elementary charge), in order to mimic the chemical bonds in the bulk of InAs WZ and ZB bulk. In addition, the atomic positions of both slabs were relaxed through DFT force and energy minimizations while keeping the lattice parameters fixed. For WZ(112̄0), only the top six layers were allowed to relax while the other four layers at the bottom were kept fixed. In the ZB(110) case, only the three topmost layers were allowed to relax. To create a vacancy, we removed either one As or In atom from the top surface of each slab before relaxation. Similarly, we substitute one As or In with one Bi atom corresponding to a Bi coverage on the surface when earlier created As/In vacancy are filled *via* Bi incorporation. The distances between each Bi atom in the WZ (ZB) structure in the *a*-, *b*-directions were approx. 7.50 Å and 7.24 Å (8.56 Å and 12.11 Å), respectively. To obtain the formation energy (*E*_f_) when Bi atoms are deposited on top or substitute As/In atoms, we calculated 
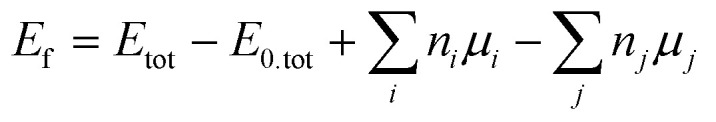
 where *E*_tot_ is the total energy of the structure with substitutional or vancancy defects, *E*_0,tot_ the total energy of the pristine structure, *n*_*i*_ the number of As/In atoms removed, *m*_*i*_ the number of Bi added, and *μ* the chemical potential of the respective atom. The chemical potential was based on Bi, and As rhombohedral phase, and In tetragonal phase structure, where for Bi, a lattice parameter of *a* = *b* = *c* = 4.79 Å was considered, for As *a* = *b* = *c* = 4.17 Å, and for In *a* = *b* = 3.18 Å and *c* = 4.85 Å. The atoms were allowed to relax until all the forces and total energy were minimized within a convergence criteria threshold of at least 10^−3^ Ry Bohr^−1^ and 10^−3^ Ry, respectively. Quantum ESPRESSO uses a plane-wave basis set to perform the DFT calculation. Therefore, we consider the GGA-PBE method to be the exchange correlation functional with a plane-wave cut-off of 60 Ry for the ultrasoft norm-conserving non-relativistic RRJK pseudopotential.^28,29^

## Results and discussion

As the first step of the studies, the native oxide was removed from the InAs surfaces using atomic hydrogen cleaning^30^ to establish identical starting conditions in all experiments. This leads to ordered, crystalline and unreconstructed surfaces for the InAs.^16^ To tune the Bi incorporation on the InAs surfaces, we followed two different thermal routes for the experiments (Fig. 1c), potentially taking advantage of kinetic effects: Bi was deposited onto InAs either at room temperature (RT) or elevated temperature (290 °C), and in both cases the effects of subsequent annealing up to 400 °C were investigated. After each step, XPS data was obtained. Measurements were carried out on ensembles of nanosheets deposited on a standard Si substrate (with a thin native Si-oxide) and InAs(110) substrates. Additional nanoscale spatially resolved XPS measurements were performed for selected deposition steps using XPEEM and SPEM. Further details can be found in the Experimental section.

### Bi deposition on InAs surfaces and Si substrates

Initially, we directly compare the amount of Bi adsorbed on the InAs sheets with that on the Si substrate. The Bi 4f signal was measured in areas with/without InAs nanostructures by moving the sample in the X-ray beam. We find that the sticking probability of Bi atoms on the Si substrate must be significantly lower than on the InAs nanosheets for all depositions. No Bi 4f signal could be detected on the Si regions without InAs nanosheets for most deposition and annealing steps. Only after the longest RT deposition and the first following annealing, Bi was found on bare Si. The ratio between Bi on Si and on the InAs nanosheets is highest directly after this last deposition with about twice as much Bi sticking to the InAs sheets (see section S1 in the ESI†). This is an interesting result as it shows that Bi can be selectively added to an InAs device component on a standard Si substrate. It allows for manipulation of InAs devices with Bi after fabrication on the Si platform. Further, it shows how the Bi signals discussed below can be directly related to the sheets, simplifying the interpretation. To also compare the amount of Bi adsorbed on the InAs WZ nanosheet *versus* the ZB substrate we used XPEEM. Nanosheets placed on a InAs(110) substrate were imaged after deposition of Bi at elevated temperature. This allows us to measure XPS from the two different areas of the sample (as shown in Fig. 1d). Here, the amount of Bi adsorbed on the InAs substrate is 2.5 times larger than on the InAs sheets.

### Room temperature deposition on WZ(112̄0)

The following results were obtained with nanosheets deposited on Si substrates as mentioned above.

**Fig. 2:**
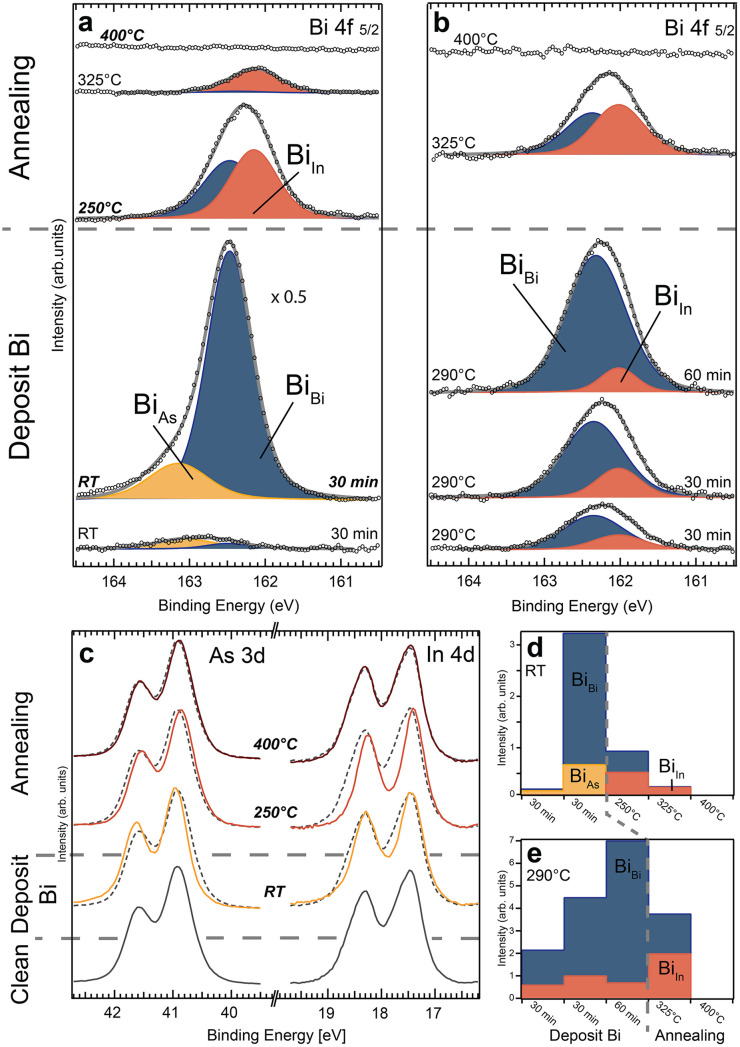
Bi 4f 5/2 core level spectra from the InAs WZ(112̄0) nanosheets deposited on Si wafers for subsequent deposition and annealing steps (each 10 min long). The substrate temperature was left at room temperature (a) or heated to around 290 °C (b) for each deposition. The intensity for the 2^nd^ deposition step in (a) was multiplied by 0.5 for better comparison. Depending on the deposition and annealing parameters three different components are present (Bi_Bi_, Bi_As_ and Bi_In_) on the WZ(112̄0) crystal facet. We denote Bi_Bi_ to metallic Bi bonds. For further details see the text. (c) As 3d and In 4d core level spectra for selected process steps in (a) indicate distinct shifts towards higher and lower BE, respectively. The signal of the clean InAs surface is superimposed as a dashed line for each process step and the intensities have been scaled for better comparison. (d) and (e) show the integrated area of each component peak for the process sequences in (a) and (b), respectively.

In the first deposition scheme, Bi was evaporated onto the InAs sheets at RT in two steps and subsequently annealed to higher temperatures. Fig. 2a shows the acquired XPS spectra of the Bi 4f 5/2 core level peak after each step. The spectra show up to three different Bi components (Bi_Bi_, Bi_As_ and Bi_In_), corresponding to different Bi binding configurations. The fitting of the core level spectra is described in the experimental and computational details section. Two peak components (Bi_Bi_, Bi_As_) are visible after both RT deposition steps. Both increase with deposition time. However, Bi_Bi_ becomes much larger than Bi_As_ after the second deposition. As it is known that metallic Bi layers grow on the InAs surface at RT, we can attribute the main component Bi_Bi_ after the second deposition (over 1.5 monolayers (MLs) deposited) to metallic Bi forming on the surface. The binding energy (BE) of Bi_As_ in all spectra discussed below is 0.56 ± 0.06 eV higher than the metallic Bi_Bi_ component. Due to the enhanced electronegativity of As compared to Bi, we attribute it to Bi bonding primarily to As surface atoms, in agreement with literature.^26^ We can thus see that the initial deposition is dominated by bonds between Bi and surface As atoms followed by Bi–Bi bond formation on top. For additional information, As and In core level XPS spectra were measured (see Fig. 2c and S2 in the ESI†). For As 3d (In 4d), the surface component is located at lower (higher) binding energy compared to the bulk peak.^21,31^ The evolution of components detected in the Bi 4f spectra in Fig. 2a coincides with shifts detected in the As 3d and In 4d spectra (Fig. 2c), respectively. We observe a shift towards higher binding energy of the As 3d peaks, which is consistent with As–Bi bonding visible in the Bi spectra after the depositions and a reduction of the surface component (at lower BE). The In 4d spectra also show a lowering of the surface component, which agrees with Bi placed on top of an InAs surface. Even if bonding occurs primarily between Bi and As, the surface In atoms should still be influenced, as is observed. In summary, we interpret these spectra as Bi initially adsorbing on the unreconstructed InAs surface, mostly bonding with As surface atoms. The metallic component appears before a full ML of Bi has been grown indicating that island growth of Bi layers occurs.

Subsequent annealing of the sample to 250 °C and then to 325 °C gives rise to another component (Bi_In_) in the Bi 4f spectra. The BE position in all analysed spectra is 0.29 ± 0.04 eV lower compared to metallic Bi. We attribute Bi_In_ to Bi–In bonds based on the lower electronegativity of In compared to metallic Bi.^26^ Concurrently, the Bi–As component vanishes and the metallic Bi signal decreases significantly, resulting in a decrease in the total amount of Bi. Annealing from 250 °C to 325 °C decreases the signal of Bi–In bonds by ∼50% whereas the metallic Bi signal reduces to 10% of its previous size indicating that most of the metallic Bi vanishes from the surface (Fig. 2a and d). This shows that a significant amount of the RT deposited Bi desorbs while some Bi atoms move into the new binding configuration with In during the first annealing. It also suggests that the formation of Bi–In bonds requires an additional activation energy compared to the initial on top adsorption. The change in the Bi 4f spectra coincides with a significant additional BE shift in the In 4d and a reversed shift of the As 3d core level (see Fig. 2c and S2 in the ESI†). We interpret this as a substitutional incorporation of Bi into As sites, surrounded by In atoms leading to Bi–In bonds. Such an anion exchange reaction in III–V materials is commonly observed^32^ and was, *e.g.* identified for incorporated Bi atoms on GaAs nanowires with WZ segments.^33^ Overall, this would shift the spectrum from V-Bi bonds to III-Bi bonds, as seen in our experiments in Fig. 2 for higher sample temperatures. Finally, annealing to 400 °C completely removes all Bi. Simultaneously, the As 3d and In 4d core level spectra revert to their original shape and position found immediately after removing the native oxide. This transition indicates that the top layer with In and Bi must be completely removed, and we are again left with an unreconstructed InAs surface.

In summary, three stages are observed: (i) Bi–As (Bi_As_) bonds and Bi–Bi (Bi_Bi_) bonds are detected after RT deposition, indicating Bi adsorbing on top of the surface in the vicinity of As atoms, (ii) Bi–In (Bi_In_) bonds appear upon annealing which is consistent with a thermally activated substitution of Bi into As surface sites. Additionally, Bi atoms evaporate leading to less metalling Bi and no observation of Bi–As bonds. (iii) Above 400 °C, all Bi atoms desorb completely from the WZ surface which returns to its initial state.

### Deposition at elevated temperature on WZ(112̄0)

As a second thermal route, Bi was deposited at an elevated sample temperature. Bi 4f core level XPS was again measured and analysed at each preparation step as shown in Fig. 2b. For depositions at 290 °C, the Bi–As bond configuration (component Bi_As_) is not observed. Interestingly, metallic Bi (Bi_Bi_) formation is strongly favoured over Bi–In alloying (Bi_In_). 75% of the adsorbed Bi is metallic compared to 25% during the first RT deposition. The amount of Bi–In bonds is almost constant for all deposition steps, while the signal for metallic Bi is continuously growing. This indicates a self-limiting process in which a certain Bi integration in the outermost layer of InAs can be achieved. After reaching this critical point, additional depositions will produce metallic Bi on top. As a result, sharp interfaces between the bulk InAs and the InAs:Bi compound, as well as the adsorbate layer consisting of metallic Bi, can be realized. With the increased sample temperature, the amount of adsorbed Bi is lower by roughly one order of magnitude compared to RT depositions. This indicates that significant Bi desorption occurs simultaneously with the adsorption during deposition limiting the amount of Bi on the surface. Therefore, the time of each evaporation step is extended to 30 min for the first two depositions and 60 min for the last one. This allows us to easily identify the metallic Bi component and (if present) additional features in the spectra. The subsequent annealing step to 325 °C reduces the total Bi signal by ∼30%. An additional 25% is rearranged to form Bi–In bonds (Bi_In_). The In 4d and As 3d core level spectra (shown in Fig. S3 in the ESI†) confirm these results. There is no indication of As–Bi type bonds after any process step. Similar to the first deposition and annealing cycle discussed above, no Bi is left on the surface when finally annealing the sample to 400 °C.

The reproducibility of the observed Bi bonding for individual sheets is discussed in section S4 of the ESI.† We investigated individual nanosheets for different deposition and annealing steps with SPEM. An X-ray beam spot focused down to 120 nm allowed us to measure spatially resolved XPS from individual nanosheets (details can be seen in section S3 and 4 the ESI†). The In 3d and As 4d core level spectra show a similar temperature dependency as described above. The formation of on top Bi–As bonds is favoured for lower sample temperatures. To enable Bi–In bonds sufficient energy must be supplied *via* sample heating.

### Room temperature deposition on ZB(110)

**Fig. 3:**
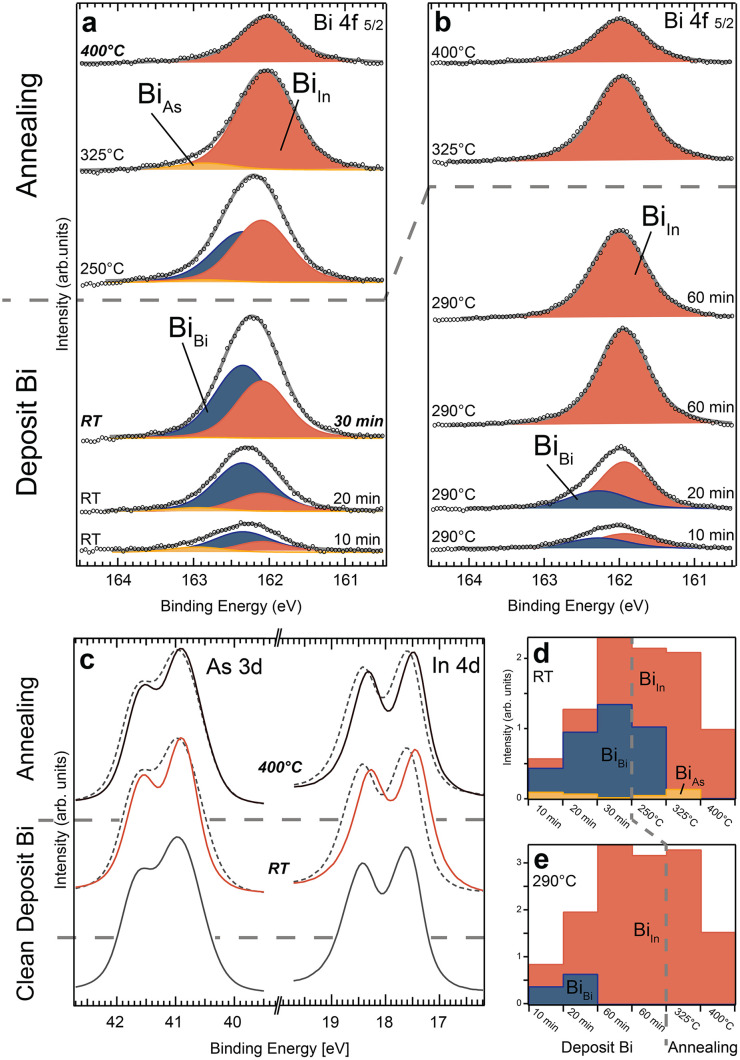
Bi 4f 5/2 core level from the InAs ZB(110) substrate for all deposition and subsequent annealing steps (each 10 min long). The substrate temperature was left at room temperature (a) or heated to around 290 °C (b) for each deposition. Three components (Bi_Bi_, Bi_As_ and Bi_In_) similar to the Wz(112̄0) crystal facet are detected. For further details see the text. (c) As 3d and In 4d core level spectra for selected process steps in (a) indicate mainly a reduction in bulk and surface component for As and a distinct shift for In due to the formation of an In–Bi component at lower binding energies. The signal of the clean InAs surface is superimposed as a dashed line for each process step for better comparison and the intensities have been normalized. (d) and (e) show the integrated area of each component peak for the process sequences in (a) and (b), respectively.

Moving on, we investigate Bi deposition on InAs ZB(110) bulk substrates with XPS results shown in Fig. 3. When depositing at room temperature, we find significant differences compared to the WZ(112̄0) surface. The In 4d spectra of the InAs(110) surface show a distinctive shift towards lower binding energies for all process steps, which indicates a reduction of the In surface component and instead an increase of a new component at lower BE (see Fig. 3c and the ESI† for curve fitting). This we interpret as an In–Bi bond configuration. In contrast, there is no clear indication of As–Bi arising in the As 3d spectra (as compared to the spectra of WZ(112̄0)). For the Bi 4f 5/2 core level, we find that the metallic Bi (Bi_Bi_) bonds dominate during all deposition steps at RT (Fig. 3a and d). However, the amount of Bi–In is steadily increasing, shifting the ratio between Bi–In and Bi–Bi from 1 : 2 for the first to a 1 : 1 for the last deposition. After the final deposition, we estimate a total Bi coverage on the surface of about two MLs, as discussed in the ESI.† A small amount of Bi–As is also noticeable in most Bi 4f spectra. During the first deposition, 15% of the Bi atoms bond primarily to As atoms (Bi_As_), decreasing for the following steps to below 6%. Similar to the WZ(112̄0) facet, annealing to 250 °C and 325 °C shifts the Bi 4f level additionally to lower binding energies. This is due to increasing amount of Bi–In bonds and vanishing metallic Bi.

In contrast to the WZ crystal facet, even high-temperature annealing to 400 °C for 10 min does not remove all Bi from the surface. Roughly half a monolayer is still present. Overall, we identify only two regions: (i) Bi–In and Bi–Bi are primarily formed after RT deposition, and (ii) for high temperature annealing up to 400 °C, metallic Bi is significantly reduced and eventually vanishes, while additional Bi–In bonds are formed. The formation of Bi–As plays only a minor role and is much smaller than Bi–In. The surface structure of InAs(110) after Bi deposition at RT and subsequent annealing has already been investigated before^34,35^ as discussed in detail in section S7 in the ESI.† While several species of Bi have also been observed in those studies similar to our case, a more elaborate Bi structure found at high temperature is not consistent with our results.

### Deposition at elevated temperature on ZB(110)

The behaviour of Bi deposited on InAs(110) at an elevated temperature is comparable to the case of WZ(112̄0). Fig. 3b and e show that only Bi–In and metallic Bi bonds are created. Also, the In and As spectra (see section S6 in the ESI†) indicate no bond formation involving As atoms. During the first deposition, the amount of metallic Bi and Bi–In is about equal based on the Bi 4f core level. This decreases for the second deposition with only 1/3 of the Bi signal corresponding to metallic Bi. Thereafter, Bi is only binding with In atoms.

Furthermore, for the two 60 min long depositions, a steady state is reached where the overall amount of Bi no longer increases. Therefore, we conclude that a comparable number of Bi atoms is leaving the surface while new ones arrive. Similar to the WZ(112̄0) crystal, Bi–In is stable for sample temperatures up to 325 °C. However, we do not see the same immediate and drastic decomposition around 400 °C, but rather a slow decrease of the Bi–In compound with a result comparable to the deposition sequence at RT.

In general, a self-limiting Bi incorporation solely in the top surface layer as detected on WZ(112̄0) does not exist for ZB(110). Instead, an increase of Bi–In bonds in the Bi 4f and In 4d core level spectra is observed for each deposition step. Thickness estimations suggest about two MLs of Bi deposited at the end of the evaporation sequence. Therefore, sub-surface layers need to accommodate Bi atoms in order to account for the large amount of Bi–In bonds.

### Theoretical calculations

To better understand Bi adsorption on each InAs surface, DFT calculations were performed (described in detail in the experimental and computational details section). For both crystal phases, we focus on two specific scenarios: (i) Bi adsorbing on top of the pristine InAs, and (ii) In (or As) surface atom substitution *via* a single Bi atom.

Relaxing a pristine InAs crystal without any Bi atom results in a buckled top layer for both, WZ(112̄0) and ZB(110) facets. In general, As atoms are protruding further out of the crystal, while In atoms from the top layer are being pushed towards the second layer (see section S9 in the ESI† for illustrations of all scenarios).

**Fig. 4:**
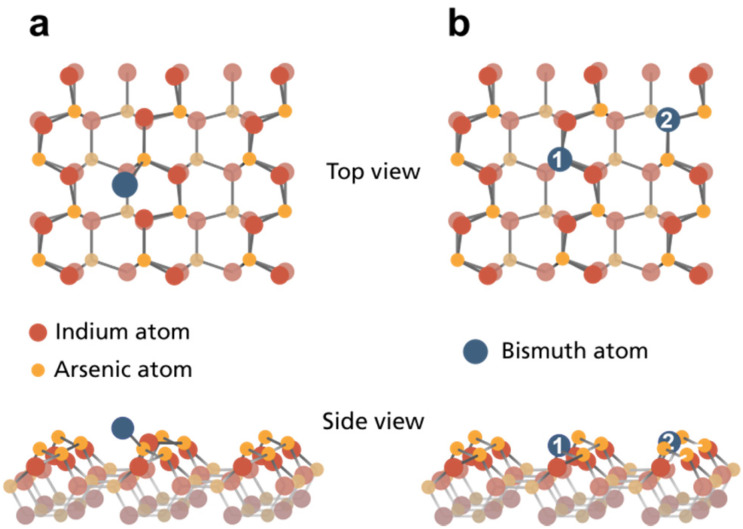
Ball model of the InAs WZ(112̄0) surface structure with (a) the most favorable bridge position for Bi atoms adsorbed and (b) substitution of As (1) and In (2) surface atoms based on DFT calculations.

Considering case (i) with Bi adsorbing on top of the pristine InAs crystal, the formation energies for WZ(112̄0) and ZB(110) are very similar (0.696 and 0.633 eV, see Table 1). Fig. 4a displays, the most stabile bridge position of a Bi adatom on WZ(112̄0) closest to an As surface atom consistent with the observed Bi–As bond component in our experiments.

**Table 1:** Formation energies for surface processes based on DFT calculations. The energies are per unit cell

Energy needed [eV]:	WZ(112̄0)	ZB(110)
As vacancy formation	1.255	1.255
In vacancy formation	1.629	1.840
Bi substitution with As	0.185	0.308
Bi substitution with In	0.325	0.297
Bi on top adsorption	0.696	0.633

Moving towards incorporating Bi, we see that the surface buckling is not significantly altered by creating In or As vacancies. Here, only the nearest neighbouring atoms move a bit further down. The calculated energies for both crystal surfaces and vacancy types can be found in Table 1 indicating that endothermic reactions are necessary to pull out any surface atom. By filling these vacant sites with Bi atoms, we obtain case (ii) (see Fig. 4b for WZ). The surface buckling vanishes within the vicinity of the Bi atom for both crystal facets. However, additional surface modulation occurs due to the larger size of the adatom. The calculations show that the modulation height of both crystals increases to 0.40 Å for InAs WZ(112̄0) and 0.27 Å for ZB(110) surfaces. For InAs WZ(112̄0), the most stable configuration corresponds to Bi substituting As with a formation energy of 0.185 eV per slab super-cell. Conversely, In or As substitution by Bi in the InAs ZB(110) surface have similar formation energies of approx. 0.30 eV.

It should be noted that our calculations show that surface atom substitution by Bi is more favourable than an on top Bi adsorption for both investigated systems. This explains the appearance of the In–Bi species at higher temperatures and supports that it must be a substitutional incorporation (as the Bi surface positions near In are less favourable on top of the unreconstructed surface). The reason for the species not appearing immediately on the WZ surface can be explained by the presence of a kinetic barrier for substitution as have been seen for other III–V systems.^36^ We can rule out that the transition occurs through an initial, separate vacancy formation as this is energetically much less favourable. Instead, a concerted exchange of a Bi atom with an As atom takes place, similar to other anion exchange reactions.^36^ That it occurs already at RT on InAs ZB(110) is in agreement with previous observations of RT exchange reactions at low temperatures in other III–V systems.^37^ We do not observe Bi substituting In in the surface although this should be equally favourable on the ZB surface, but rather interpret the low amount of Bi–As detected to filling of already existing In vacancies in the surface. We thus attribute this again to kinetic barriers, and it could explain the differences seen in a few other studies of InAs(110).^34,35^ Additionally, morphological differences can also play a role in the incorporation as substitution could be initiated at surface steps where kinetic barriers can be lowered. While this has been observed for some systems, we have no indication for this in the present case. Finally, the calculations explain the stability at higher temperatures of the In–Bi structures on ZB(110) compared to WZ(112̄0). The formation energy for Bi substitution of As is significantly higher on ZB(110). Therefore, it will take significantly more energy to remove the substituted Bi atom from the ZB surface leading to the observed Bi spectra at 400 °C. This is surprising considering that both InAs crystal surfaces have the same nearest neighbour configuration. Therefore, we can conclude that the overall arrangement of the lattice (beyond the direct neighbouring atoms), either in a zigzag (ZB) or armchair (WZ) geometry, has a strong impact on the adsorption and incorporation of Bi atoms.

## Conclusions

We investigated the incorporation of Bi adatoms into InAs nanosheets with a WZ(112̄0) lattice in comparison to a bulk ZB(110) surface. A dependency, for the formation of Bi–As and Bi–In bonds, on the sample crystal facet was found. The bond formation on the ZB and WZ crystal was similar for depositions at elevated temperatures but showed distinct differences when the sample was left at room temperature. Generally, a thin film formation is observed on both InAs surfaces. The first Bi adatoms on the surface will bond either on top as with the As–Bi or substitutional with the In–Bi bonded species upon which a Bi metal layer will grow. Our measurements show a self-limitation of surface alloying of Bi atoms into the WZ(112̄0) surface for both experimental sequences. A clear dominance of the metallic Bi component is seen after each final deposition step (see Fig. 2d and e) with over a monolayer coverage. This indicates a single atomic layer interface between the deposited Bi layer and the original InAs surface. These findings are in contrast to the InAs ZB(110) surface. Here, Bi substitutes As in the surface already at RT. Furthermore, Bi diffuses into sub-surface layers inhibiting the formation of an atomically sharp transition to InAs. Thus, we can conclude that chemically very different interfaces can be constructed depending on the detailed crystal structure with more control available on the WZ nanosheet.

This study represents the first holistic chemical analysis of Bi induced surface changes in an InAs WZ crystal. Interestingly, in a direct comparison between InAs nanosheets and a Si substrate, we see that the probability of Bi adatoms sticking on a native silicon oxide is significantly lower for all process steps essential for device fabrication. Additionally, the newly formed surface layer exhibiting Bi–In bonds is stable for temperatures up to 325 °C. The formation of exclusive Bi–In bonds upon Bi deposition is highly promising for the formation of heterostructures with a potential type-II Dirac and topological nodal line semimetal.^38^ Our results give a clear indication that a door towards novel nano-engineered devices based on core–shell heterostructures in the field of optoelectronics and spintronics has been opened.

## Conflicts of interest

There are no conflicts to declare.

## Supplementary Material

NR-015-D3NR00454F-s001

## References

[cit1] Lutchyn R. M., Bakkers E. P. A. M., Kouwenhoven L. P., Krogstrup P., Marcus C. M., Oreg Y. (2018). Nat. Rev. Mater..

[cit2] Hrachowina L., Chen Y., Barrigón E., Wallenberg R., Borgström M. T. (2022). Mater. Today Energy.

[cit3] Zheng X., Horng R. H., Wuu D. S., Chu M. T., Liao W. Y., Wu M. H., Lin R. M., Lu Y. C. (2008). Appl. Phys. Lett..

[cit4] Tung R. T. (2014). Appl. Phys. Rev..

[cit5] Palmstrøm C. J., Cheeks T. L., Gilchrist H. L., Zhu J. G., Carter C. B., Wilkens B. J., Martin R. (1992). J. Vac. Sci. Technol., A.

[cit6] Yang F., Elnabawy A. O., Schimmenti R., Song P., Wang J., Peng Z., Yao S., Deng R., Song S., Lin Y., Mavrikakis M., Xu W. (2020). Nat. Commun..

[cit7] Liu X., Zhang S., Guo S., Cai B., Yang S. A., Shan F., Pumera M., Zeng H. (2020). Chem. Soc. Rev..

[cit8] Wei D., Maddox S., Sohr P., Bank S., Law S. (2020). Opt. Mater. Express.

[cit9] Isaeva A., Ruck M. (2020). Inorg. Chem..

[cit10] Ferhat M., Zaoui A. (2006). Phys. Rev. B: Condens. Matter Mater. Phys..

[cit11] Fang Z., Gao H., Venderbos J. W. F., Rappe A. M. (2020). Phys. Rev. B.

[cit12] Polak M. P., Scharoch P., Kudrawiec R. (2015). Semicond. Sci. Technol..

[cit13] Balanta M. A. G., Kopaczek J., Orsi Gordo V., Santos B. H. B., Rodrigues A. D., Galeti H. V. A., Richards R. D., Bastiman F., David J. P. R., Kudrawiec R., Galvão Gobato Y. (2016). J. Phys. D: Appl. Phys..

[cit14] Walther M., Schmitz J., Rehm R., Kopta S., Fuchs F., Fleißner J., Cabanski W., Ziegler J. (2005). J. Cryst. Growth.

[cit15] Dey A. W., Svensson J., Borg B. M., Ek M., Wernersson L. E. (2012). Nano Lett..

[cit16] Hjort M., Lehmann S., Knutsson J., Zakharov A. A., Du Y. A., Sakong S., Timm R., Nylund G., Lundgren E., Kratzer P., Dick K. A., Mikkelsen A. (2014). ACS Nano.

[cit17] Rota M. B., Ameruddin A. S., Fonseka H. A., Gao Q., Mura F., Polimeni A., Miriametro A., Tan H. H., Jagadish C., Capizzi M. (2016). Nano Lett..

[cit18] Ullah A. R., Joyce H. J., Burke A. M., Wong-Leung J., Tan H. H., Jagadish C., Micolich A. P. (2013). Phys. Status Solidi RRL.

[cit19] Elayech N., Fitouri H., Boussaha R., Rebey A., El Jani B. (2016). Vacuum.

[cit20] Nakamura T., Ohtsubo Y., Yamashita Y., Ideta S., Tanaka K., Yaji K., Harasawa A., Shin S., Komori F., Yukawa R., Horiba K., Kumigashira H., Kimura S. (2018). Phys. Rev. B.

[cit21] Nicolaï L., Mariot J., Djukic U., Wang W., Heckmann O., Richter M. C., Kanski J., Leandersson M., Balasubramanian T., Sadowski J., Braun J., Ebert H., Vobornik I., Fujii J., Minár J., Hricovini K. (2019). New J. Phys..

[cit22] Richter M. C., Mariot J., Gafoor C., Nicolaï L., Heckmann O., Djukic U., Vobornik I., Fujii J., Barrett N., Feyer V., Schneider C. M., Hricovini K. (2016). Surf. Sci..

[cit23] Ahola-Tuomi M., Punkkinen M. P. J., Laukkanen P., Kuzmin M., Lång J., Schulte K., Pietzsch A., Perälä R. E., Räsänen N., Väyrynen I. J. (2011). Phys. Rev. B: Condens. Matter Mater. Phys..

[cit24] Shen P. C., Su C., Lin Y., Chou A. S., Cheng C. C., Park J. H., Chiu M. H., Lu A. Y., Tang H. L., Tavakoli M. M., Pitner G., Ji X., Cai Z., Mao N., Wang J., Tung V., Li J., Bokor J., Zettl A., Wu C. I., Palacios T., Li L. J., Kong J. (2021). Nature.

[cit25] Pan D., Wang J., Zhang W., Zhu L., Su X., Fan F., Fu Y., Huang S., Wei D., Zhang L., Sui M., Yartsev A., Xu H., Zhao J., Wang J., Zhang W., Zhu L., Su X., Fan F., Fu Y., Huang S., Wei D., Zhang L., Sui M., Yartsev A., Xu H., Zhao J. (2019). Nano Lett..

[cit26] Szamota-Leandersson K., Leandersson M., Göthelid M., Karlsson U. O. (2011). Surf. Sci..

[cit27] Giannozzi P., Baroni S., Bonini N., Calandra M., Car R., Cavazzoni C., Ceresoli D., Chiarotti G. L., Cococcioni M., Dabo I., Dal Corso A., De Gironcoli S., Fabris S., Fratesi G., Gebauer R., Gerstmann U., Gougoussis C., Kokalj A., Lazzeri M., Martin-Samos L., Marzari N., Mauri F., Mazzarello R., Paolini S., Pasquarello A., Paulatto L., Sbraccia C., Scandolo S., Sclauzero G., Seitsonen A. P., Smogunov A., Umari P., Wentzcovitch R. M. (2009). J. Phys.: Condens. Matter.

[cit28] Vanderbilt D. (1990). Phys. Rev. B: Condens. Matter Mater. Phys..

[cit29] Garrity K. F., Bennett J. W., Rabe K. M., Vanderbilt D. (2014). Comput. Mater. Sci..

[cit30] Hilner E., Håkanson U., Fröberg L. E., Karlsson M., Kratzer P., Lundgren E., Samuelson L., Mikkelsen A. (2008). Nano Lett..

[cit31] Andersen J. N., Karlsson U. O. (1990). Phys. Rev. B: Condens. Matter Mater. Phys..

[cit32] Wang Y. Q., Wang Z. L., Brown T., Brown A., May G. (2002). J. Cryst. Growth.

[cit33] Liu Y., Knutsson J., Wilson N., Young E., Lehmann S., Dick K. A., Palmstörm C. J., Mikkelsen A., Timm R. (2021). Nat. Commun..

[cit34] Betti M. G., Berselli D., Mariani C. (1999). Phys. Rev. B: Condens. Matter Mater. Phys..

[cit35] De Renzi V., Betti M. G., Corradini V., Fantini P., Martinelli V., Mariani C. (1999). J. Phys.: Condens. Matter.

[cit36] Hjort M., Kratzer P., Lehmann S., Patel S. J., Dick K. A., Palmstrøm C. J., Timm R., Mikkelsen A. (2017). Nano Lett..

[cit37] Sporken R., Xhonneux P., Caudano R., Delrue J. P. (1988). Surf. Sci..

[cit38] Ekahana S. A., Wu S. C., Jiang J., Okawa K., Prabhakaran D., Hwang C. C., Mo S. K., Sasagawa T., Felser C., Yan B., Liu Z., Chen Y. (2017). New J. Phys..

