# Supplementary material for: Tuneable 2D surface Bismuth incorporation on InAs nanosheets
Source: Nanoscale. 2023 May 12;15(21):9551–9. doi: 10.1039/d3nr00454f (PMC10234255; doi:10.1039/d3nr00454f)
Supplement: NR-015-D3NR00454F-s001 [file NR-015-D3NR00454F-s001.pdf]

Electronic Supplementary Material (ESI) for Nanoscale.
This journal is © The Royal Society of Chemistry 2023

# Supplementary

## S1 Separating the contribution to the XPS signal of the Si substrate from the InAs nanosheets

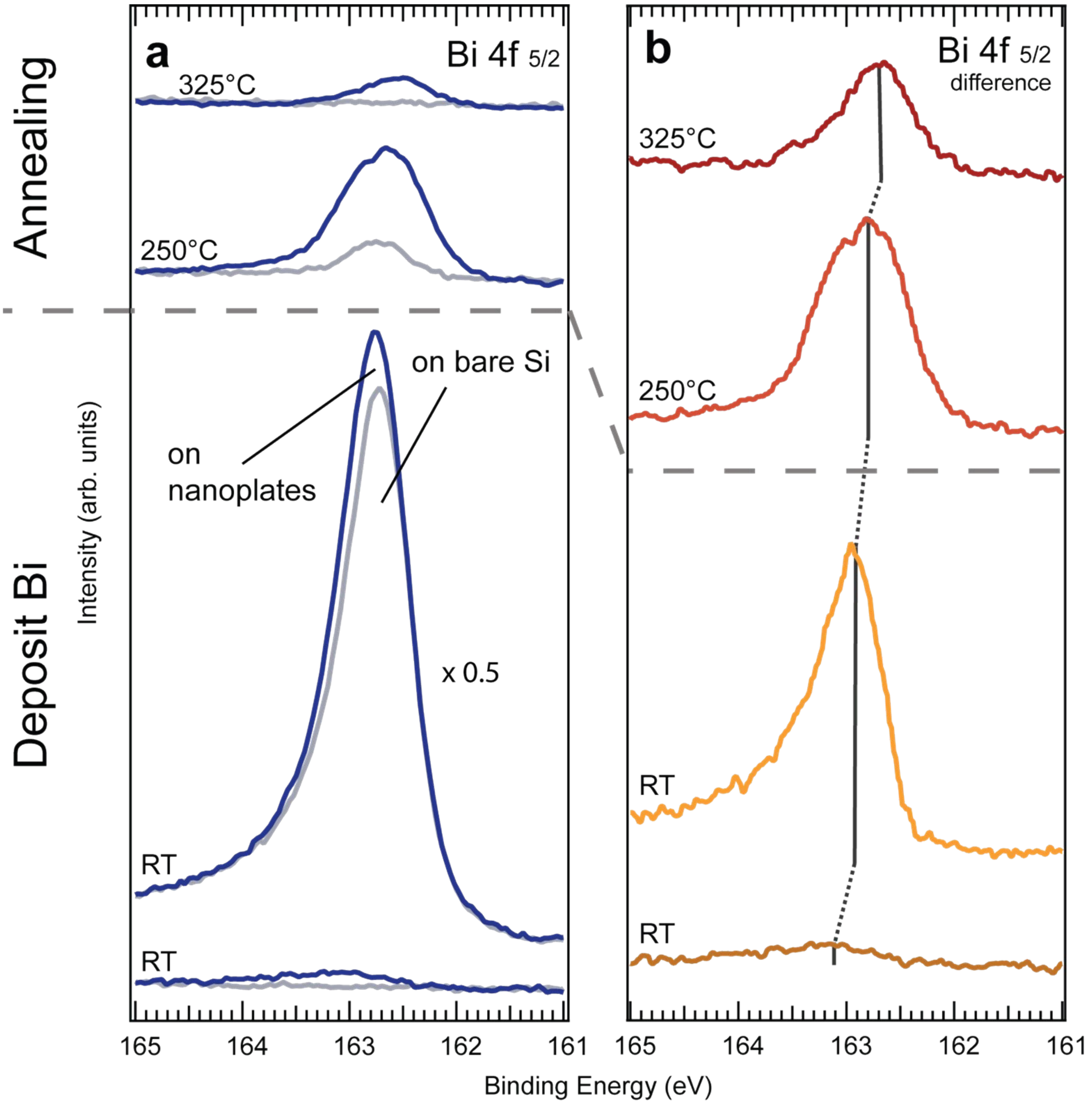


*Figure S1: (a) Bi 4f XPS signal from region filled with the InAs nanosheets (bare Si substrate) shown in blue (grey). The sample preparation consisted of two 30 min Bi depositions with the sample at room temperature and two subsequent 10 min with the sample heated to the indicated temperature for each spectra. (b) difference between both regions depicted in (a) for the respective process step. Grey line is a guide for the eye to indicate the overall peak shift to lower binding energies.*

To understand the impact of deposited Bi atoms on the InAs surface, we need to distinguish between the contribution of the Si substrate and InAs nanosheets to the Bi 4f core level signal. The X-ray beam spot for the XPS experiments was around 50x15 µm². The same core levels were recorded at a position on the InAs nanosheets and ~2 mm away from the region with the

nanostructures. For most depositions and annealing steps no Bi could be observed on regions with no nanosheets. On bare Si, a Bi signal was visible only for the second deposition at room temperature and the first annealing step (see figure S1b). After correcting the shift of each spectrum based on the Si 2p signal for each spectrum, the signal from the bare silicon was subtracted from the signal recorded on the nanosheets. The resulting datasets solely originating from the InAs nanosheets are plotted in figure S1a.

The signal of Bi on the area with bare Si was 20% and 80% smaller than on the InAs nanosheets for the large RT deposition and the following annealing step, respectively (figure S1b). By comparing the attenuation of the Si 2p spectra in areas with/without the nanosheets, we can further estimate how much of the surface is covered by the sheets (as the sheets are 20nm thick, no Si signal will be derived from the covered area). For the measurements with the largest Bi coverage, this number is 30%. Thus, assuming a similar sticking of Bi on the Si in the areas with/without plates, this leads to the conclusion that the amount of Bi on the sheets must be ~2 times larger than on the bare Si. Further, it is evident that for all process steps the overall peak in figure S1a is continuously shifting towards smaller binding energies with an overall shift of around 500 meV. That corresponds well with the fitted components in figure 1 (the untreated Bi XPS spectra). For the first deposition, Bi bonds predominantly to As atoms in the surface. This component is at higher binding energies compared to metallic Bi bonds, which are also present on the surface. After the second deposition, Bi-Bi bonds dominate over As-Bi which results in a shift towards lower energies. However, the shoulder on the left side of the peak is a strong indication that also the number of bonds between different elements of group V increases. For both subsequent annealing steps, the peak position shifts even further to the right. This is a consequence of disappearing bonds between group V elements and the formation of new In-Bi bonds. In particular, As-Bi bonds completely vanish after exposing the sample to elevated temperatures, and the amount of metallic Bi strongly decreases. Overall, the shift in the Bi 4f core level in figure 2a towards lower binding energies can be attributed to the signal coming from the InAs nanostructures. It is a result of the formation of As-Bi bonds after deposition and In-Bi after annealing, respectively.

## S2 In 4d and As 3d core levels for both datasets of the InAs WZ(11-20) nanosheets on Si substrates

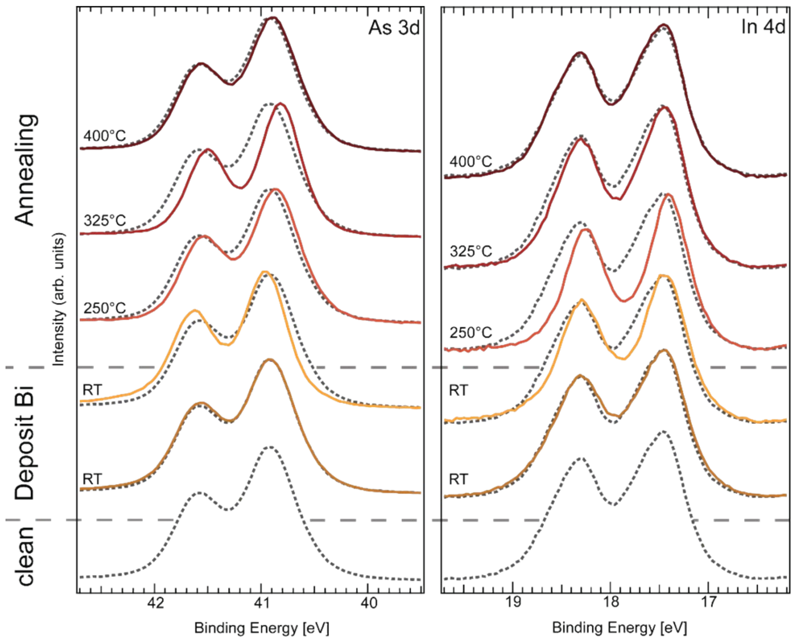


*Figure S2: (left) In 4d and (right) As 3d core level spectra of InAs nanosheets corresponding to the dataset presented in Figure 2a for depositions performed at room temperature. The signal of the clean InAs surface is superimposed for each process step for better comparison. For both core levels the intensity of individual spectra has been scaled for better comparison and a shift correction was performed based on Au 4f reference peaks. Employed photon energy 140 eV (As) and 120 eV (In).*

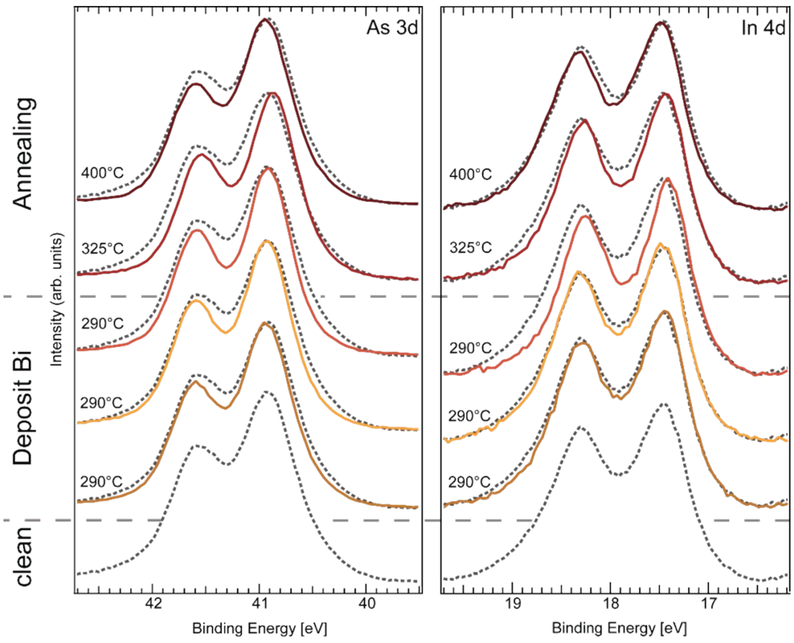


*Figure S3: (left) In 4d and (right) As 3d core level spectra of InAs nanosheets corresponding to the dataset presented in Figure 2b for depositions performed at elevated temperature. The signal of the clean InAs surface is superimposed for each process step for better comparison. For both core levels the intensity of individual spectra has been scaled for better comparison and a shift correction was performed based on Au 4f reference peaks. Employed photon energy 140 eV (As) and 120 eV (In).*

## S3 Fitting of the In 4d and As 3d core level spectra

To further verify the presence of Bi bond in the InAs surface, fits of the As 3d and the In 4d core level spectra are performed after each process step. In figure S4, we present fits for the As 3d core level on the WZ(11-20) and the In 3d core level of the ZB(110) crystal facet after the final deposition at room temperature, respectively. These datasets display the largest amount of Bi bonded to As and Bi bonded to In respectively and are therefore good for establishing the peaks related to As and In bonding to Bi. The fits were performed as stated in the Method section. For the As 3d core level spectra, the surface component appears at lower binding energies compared to the bulk doublet as can be seen in the fit of the spectra for the clean surface (and known from previous work [1]. After depositing Bi, the shift in the overall core level spectra can only be explained by the presence of another component in agreement with the As-Bi bonds detected in the Bi 4f core level in figure 2a in the main text. Furthermore, the reduction of the surface component indicates that several As surface atoms need to change their bonding configuration and bond to Bi atoms on top. In the In 4d spectrum, the surface component is situated at higher BE compared to the bulk [2]. Here a shift of the core level spectra towards lower BE is detected after the Bi deposition. To fit this spectrum, we observe a reduction of the surface component and a rise of a third doublet at lower binding energies which we attribute to In-Bi bonds in accordance with the In-Bi bonds detected in the Bi 4f spectra in figure 3a in the main text.

As can be seen, the As-B and In-Bi bonding components for both core levels are in close proximity to the respective bulk and surface components and overlap significantly. E.g., the In 4d signal on the WZ(11-20) facets of the nanosheets display a strong reduction of the surface component after the 2nd Bi deposition at room temperature and following annealing step (see figure 2c or S2). By comparing with components present in the Bi 4f fit for the respective process step, we can conclude that the In surface atoms form bonds with the arriving Bi in agreement with a gradual shift towards lower BE in the In 4d core level. However, a deconvolution of the bulk, surface and Bi-bonding component lead to higher uncertainties compared to the Bi 4f spectra. Therefore, we base our quantitative estimates of adsorption in the main text solely on the fits of the Bi 4f core level to.

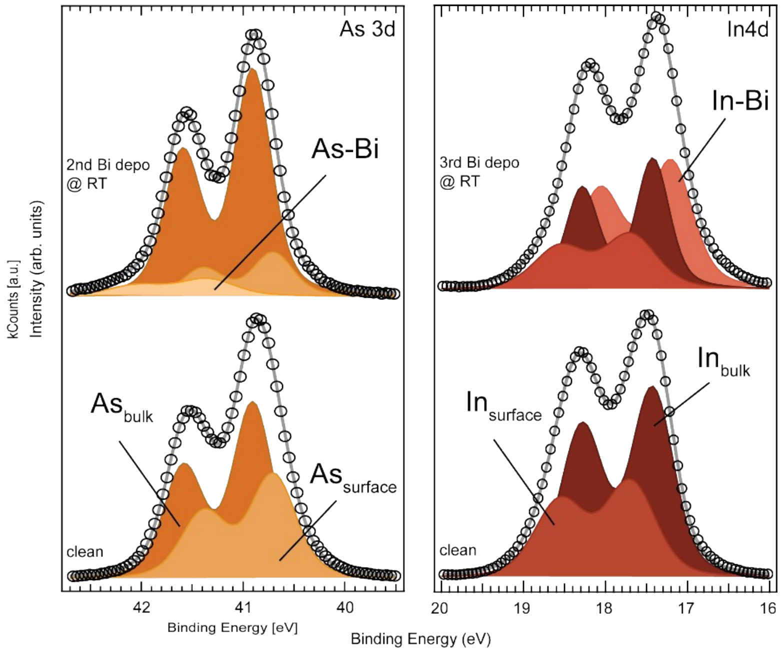


*Figure S4: fitted As 3d (on WZ(11-20)) and In 4d (on ZB(110)) core level spectra corresponding to the spectra for the respective surface after removing the oxide and the final Bi deposition at room temperature. For more details see the text.*

## S4 Procedure to find and investigate specific nanosheets in MaxPEEM and ESCA microscopy

To analyse the same nanosheets after each individual process step, a squared metal pattern consisting of rows and columns distinguishably marked with letters and numbers was created on the substrate surface via e-beam lithography. Next, nanosheets were transferred from the growth substrate to the sample by sweeping with a tailored piece of cleanroom paper over one after the other. This method enables a good distribution of transferred nanosheets. To determine suitable specimen, the surface is mapped via secondary electron microscopy as seen in figure S4. This enables us to find the same nanostructures at the MaxPEEM (MaxIV, Lund) and the ESCA Microscopy beamline (Elettra, Trieste) using the element specific-imaging mode of both setups.

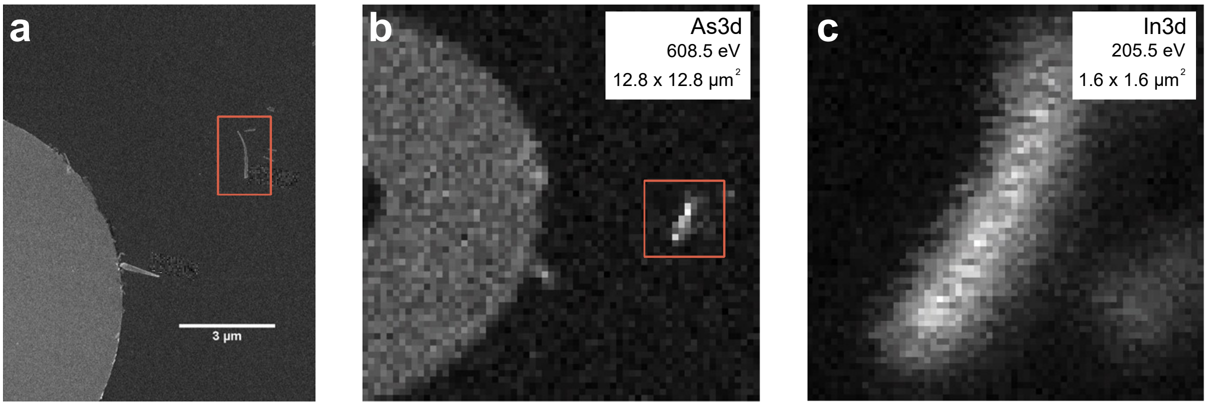


*Figure S5: procedure to map and analyze distinct InAs nanosheet with XPS: (a) determine suitable nanostructure via SEM prior to measurements. The half sphere on the left side belongs to an Au marker on the silicon substrate. (b) As 3d core level map of the position indicated in (a) with a kinetic energy of 608.5 eV. The same nanostructure is marked in both images. (c) close up of the InAs sheet by mapping the In 3d core level with a kinetic energz of 205.5 eV. The image size of (b) and (c) is indicated in the inset, repsctively.*

## S5 Single WZ nanosheet XPS analysis using a focused X-ray beam.

After sample preparation as described in S3, three individual InAs nanosheets with a WZ(11-20) crystal facet were analysed for a sequence of process steps (data of one is shown in figure S5). Initially atomic hydrogen cleaning was performed. Then a first 250°C deposition was done followed by a subsequent annealing step (both for 10 min). A second 300°C deposition was then performed which was 20 min long. Due to setup related restrictions in choice of photon energy in the SPEM imaging setup, the photon energy was 650 eV for all core level spectra and images in contrast to the energy of 120 and 140 eV used to measure the spectra of Fig 2 and 3. As a result, the ratio between bulk and surface components will be different for

the SPEM measurements due to the different mean free path of the electrons. While some differences occur, the In-As bulk component can be seen in both data sets ($In_A$ & $As_A$) for all process steps. As-Bi bonds ($As_C$) are evident for lower sample temperatures and not when the substrate is annealed to higher temperatures in agreement with measurements presented for an average of many flakes as in fig 2 and the fit shown in fig S4. In-Bi bonds ($In_C$) are exclusively formed for depositions at sample temperatures higher than 290°C also in agreement with the measurements of the main text. It can be mentioned that for the flakes, additionally, As-As bonds are present as a second component ($As_B$) in the As 3d spectrum at higher binding energies. And a small shoulder at higher binding energies is present for all In 3d spectra ($In_B$) which we identify as a little bit of In oxide still remaining on the nanosheet surface after the Hydrogen treatment [3]. This has been seen in previous studies for non-perfect Hydrogen cleanings. However, we are still able to observe the same temperature behaviour for Bi incorporation as presented in the main manuscript and the detected defects (metallic As and In oxide) appear not to interfere with the deposition or annealing process.

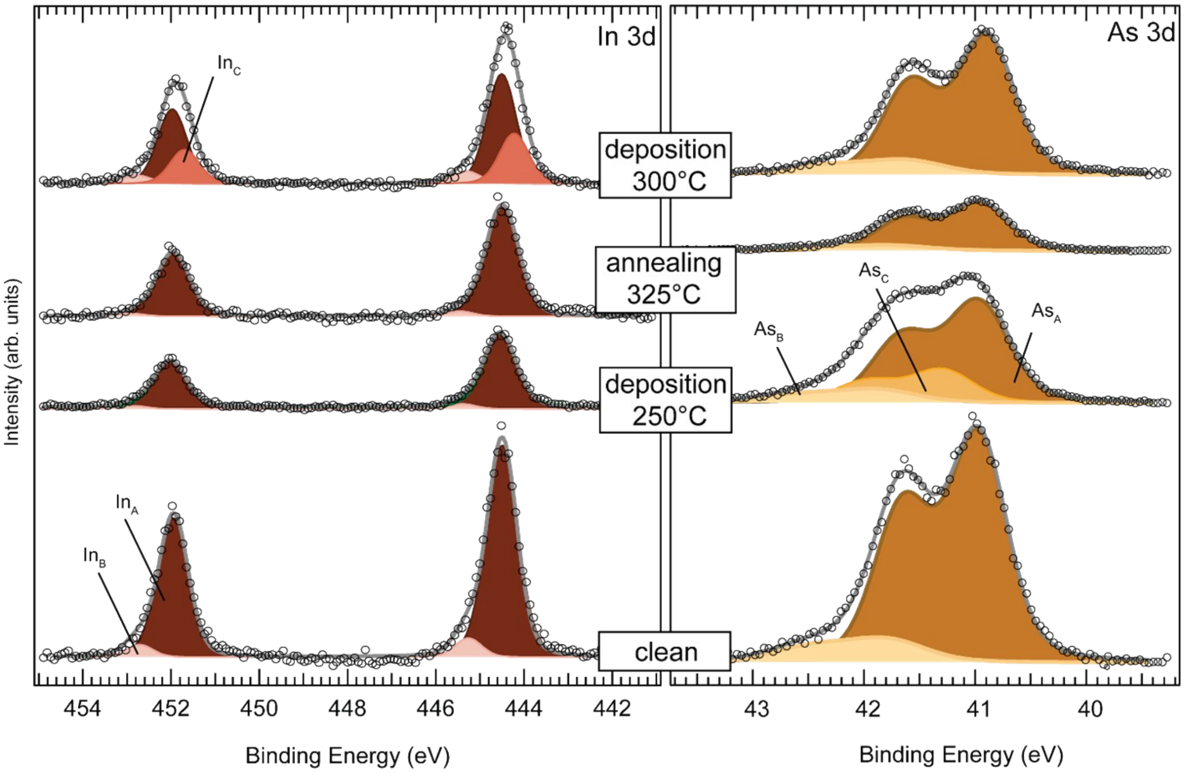


*Figure S6: XPS data for the In 3d & As 3d core level of a single nanosheet after native oxide removal and 3 subsequent preparation steps. Components binding to Bi adatoms arise only at lower sample temperatures for the As 3d core level ($As_C$), and higher temperatures for the In 3d core level ($In_C$).$In_A$ and $As_A$, as well as $In_B$ and $As_B$ refer to the bulk and defect components of the individual core level, respectively.*

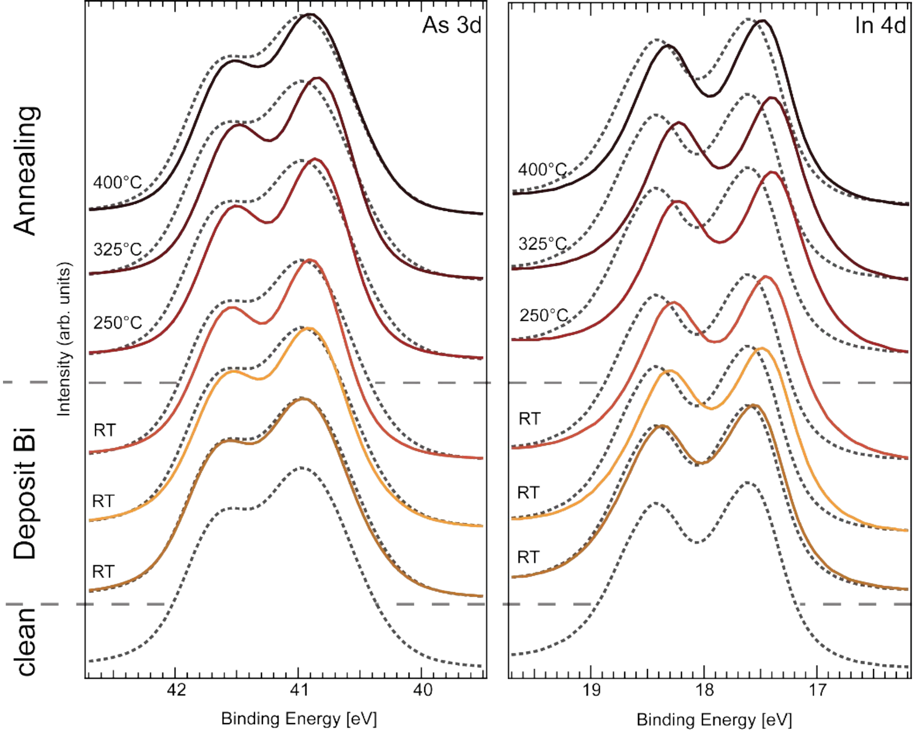


*Figure S6: (left) In 4d and (right) As 3d core level spectra of InAs Zb(110) samples corresponding to the dataset presented in Figure 2a for depositions performed at room temperature. For both core levels the intensity of individual spectra has been scaled for better comparison and a shift correction was performed based on Au 4f reference peaks. The signal of the clean InAs surface is superimposed for each process step for better comparison.*

## S6 In 4d and As 3d core levels for the InAs ZB(110) samples

Due to the significant shift of the In 4d core level to lower binding energies for all deposition and annealing steps, the fitting of the Bi 4f core level was carried out with a mandatory presence of In-Bi bonds (component $B_C$). In contrast, the As 3d core level does not reveal any additional component at higher binding energies. As a result, As-Bi bonds (component $B_B$) were only included in the Bi core level fit when a shoulder at high BEs was visible.

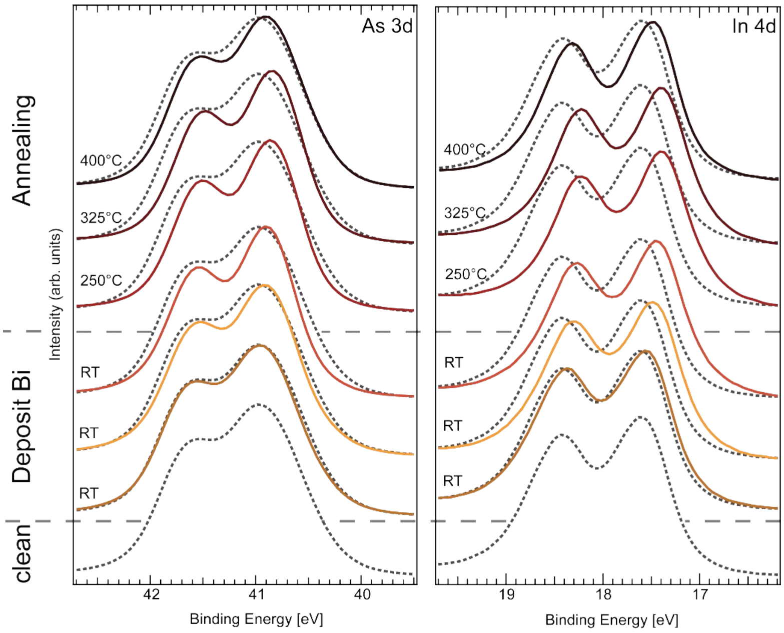


*Figure S7: (left) In 4d and (right) As 3d core level spectra of InAs Zb(110) samples corresponding to the dataset presented in Figure 2a for depositions performed at room temperature. For both core levels the intensity of individual spectra has been*

*scaled for better comparison and a shift correction was performed based on Au 4f reference peaks. The signal of the clean InAs surface is superimposed for each process step for better comparison.*

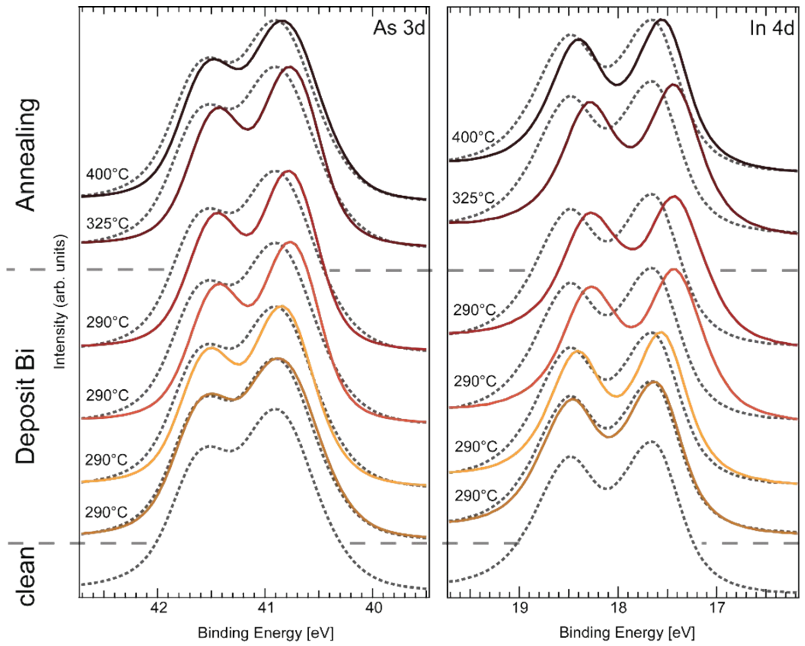


*Figure S8: (left) In 4d and (right) As 3d core level spectra of InAs Zb(110) samples corresponding to the dataset presented in Figure 2b for depositions performed at elevated temperature. For both core levels the intensity of individual spectra has been scaled for better comparison and a shift correction was performed based on Au 4f reference peaks. The dashed lines correspond to the spectra after native oxide removal.*

## S7 Model for Bi incorporation into ZB(110)

There have been several studies investigating the surface structure of InAs(110) after Bi deposition at RT and subsequent annealing steps. Renzi et al. found that during the room temperature deposition the (1x1) symmetry of the substrate is preserved and the top layer growth follows a modified Stransky-Krastanov mode [4]. Here, subsequent new surface layers start to form before the first monolayer is completed. In contrast to their work, we find that group V-Bi bonds and In-Bi bonds are present during the deposition at RT, not limited to later annealing steps, and even dominate over As-Bi bonds. However, due to the stagnating amount of As-Bi bonds and instead growth in In-Bi bonds (see figure 3d), we conclude that a layer-by-layer growth with (1x1) symmetry is not applicable in our case. Considering the scenario based solely on island growth, the vanishing surface components in the In 4d and As 3d spectra cannot be accounted for, and the modified Stransky-Krastanov growth mode is the natural choice. Subsequent annealing of the RT sample to higher temperatures will alter the surface

structure to a (1x2) reconstruction [4,5]. Earlier studies suggested a symmetry with a missing row in the second layer and followed by a tilt of the Bi atoms. As a result incorporated Bi atoms would bond among themselves and to the same type of atom in the top and second layer [5]. However, the original model indicates that As-Bi and In-Bi bonds appear with the same probability, which does not hold. Our Bi 4f spectra show a distinct shift towards lower binding energies due to the formation of additional In-Bi and the continuous disappearance of group V-V bonds. As-Bi is neglectable compared to the amount of In-Bi. Even metallic Bi vanishes above a sample temperature of 250°C disqualifying the concept of pure Bi zigzag chains along the azimuthal [1-10] direction. We therefore propose that during the high temperature annealing steps after deposition at RT, atoms originating from Bi on top either (i) substitute As surface atoms in their lattice position increasing the amount of In-Bi bonds (as depicted in figure S8) or (ii) simply desorb again from the surface. It is worth mentioning that defects in the surface like missing In atoms facilitate the formation of As-Bi to a small extent. By heating the sample to around 400°C, enough energy is supplied to the surface to cure these potential defects and a newly formed compound solely consisting of In-Bi bonds.

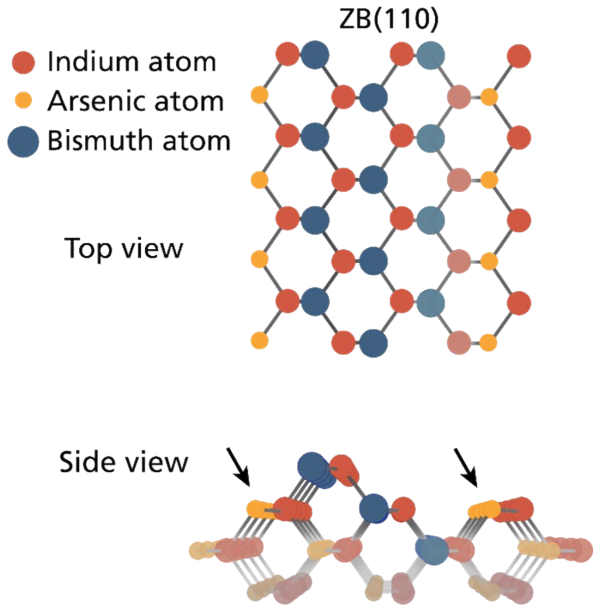


*Figure S8: Ball model of the InAs Zb(110) surface after Bi incorporation*

A similar situation evolves for depositions at higher temperatures, where we detect no As-Bi bond formation on the InAs ZB(110) surface at all. Additionally, metallic Bi seems unfavourable for long deposition times (60 min) and does not appear again for subsequent annealing steps. It is reasonable to assume that higher sample temperatures facilitate an easier

and faster substitution of As surface atoms by Bi. Additionally, theoretical studies have shown that point defects (including As vacancies) within the bulk are likely to migrate continuously towards the surface with increased sample temperature[6]. Long deposition times for samples heated to around 290°C indicate that an equilibrium state can be reached with a capping layer of more than one monolayer thickness solely exhibiting Bi-In bonds. This newly formed compound prevents further sublimation of As atoms from the original surface. However, we still see a surface component in the As 3d spectra (see figure S7 in the supplementary). Therefore, even though we expect a full coverage of the substrate with In-Bi bonds As atoms with dangling bonds must still be present. This is only possible, if instead of a (1x1) epitaxial growth a (1x2) reconstruction is considered as described above and depicted in figure S8 in the SI. Since only every second zigzag row is substituted, As atoms (indicated by the arrow in the lower part of figure S8) with surface atom characteristics remain.

## S8 Thickness estimation from XPS core level spectra

In order to estimate the thickness of the deposited Bi film, the intensity of the XPS core levels from the clean InAs substrate $I_S^0$(nanosheet or bulk) is compared with the core level intensities from the substrate after individual Bi depositions and annealing steps $I_S$ for the In 4d or As 3d core level. This allows us to calculate the thickness of the deposited film $d$ via

$$I_S = I_S^0 * exp\left( - \frac{d}{\lambda_{A,E(B)} \cos \theta} \right)$$

with $\lambda_{A,E(B)}$ being the electron attenuation length through the Bismuth layer at a specific photon energy and $\theta$ the photoemission angle with respect to the surface normal [7]. In our experiments, electrons were collected in normal emission mode ($\theta = 0°$) with a photon energy of 140 eV (120 eV) yielding an attenuation length of 6.23 Å (5.9 Å). To understand the thickness in terms of monolayers deposited, all calculated values are divided by 2.3 Å the atomic layer distance of Bi(110), where $d = 2.3$ Å corresponds to one monolayer of Bi deposited onto the sample.

S9 Crystal structures calculated by DFT for different scenarios

The color coding for the crystal structures below are as: green – Arsenic; purple – Bismuth, dusky pink – Indium.

## WZ(11-20)

| Side view | Top view |
|---|---|
| **Pristine surface** | |
| 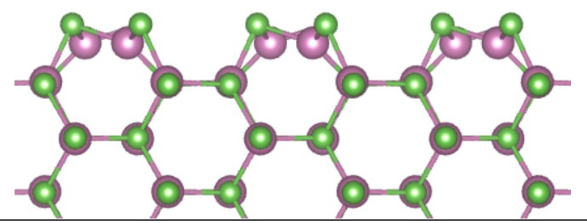 | 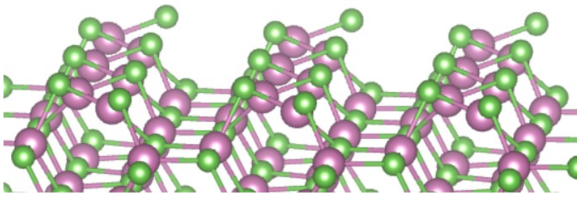 |
| **As vacancy** | |
| 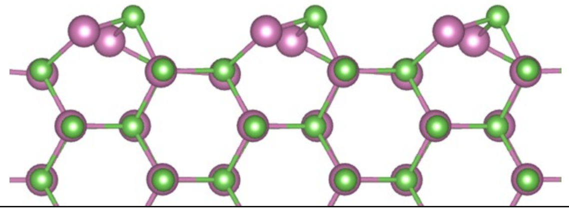 | 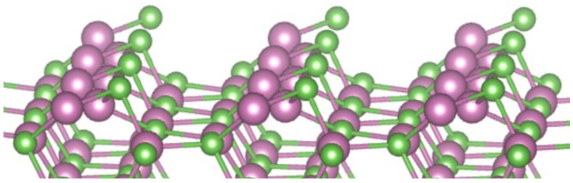 |
| **Bi substitutes As atom** | |
| 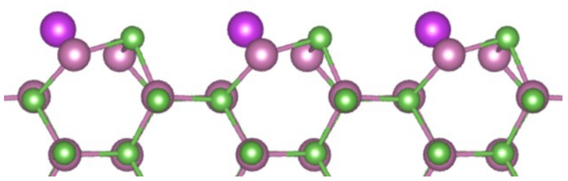 | 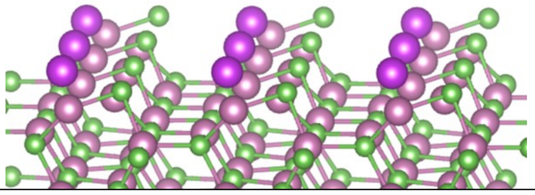 |
| **In vacancy** | |
| 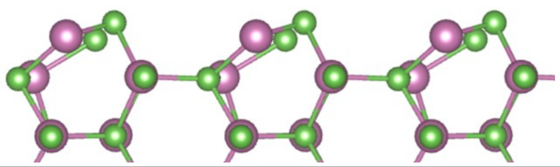 | 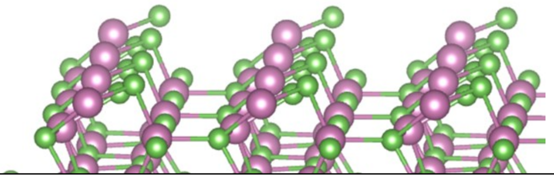 |
| **Bi substitutes In atom** | |
| 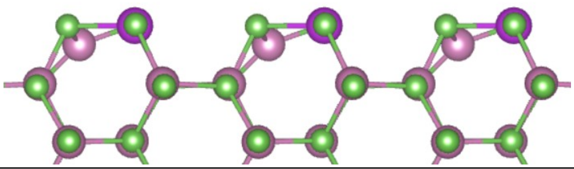 | 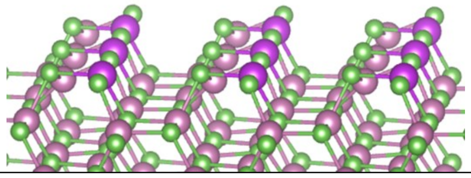 |

**ZB(110)**

| Side view | Top view |
|---|---|
| **Pristine surface** | |
| 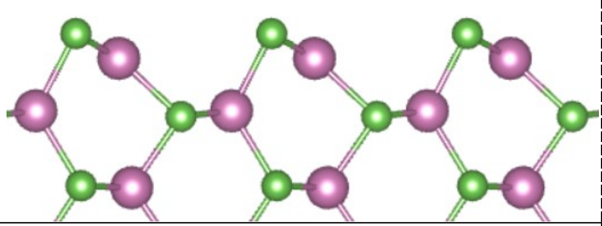 | 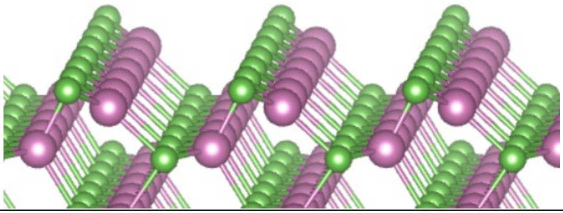 |
| **As vacancy** | |
| 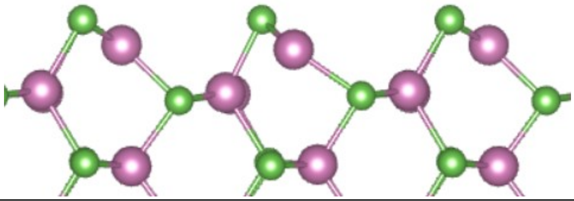 | 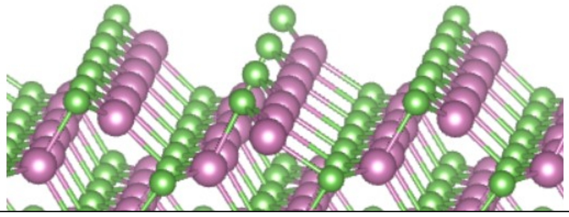 |
| **Bi substitutes As atom** | |
| 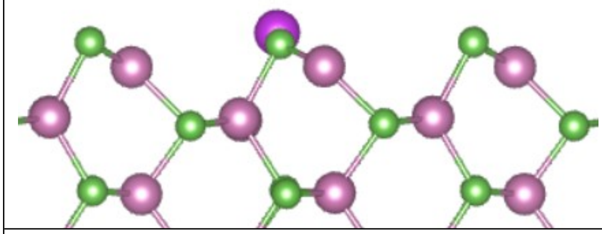 | 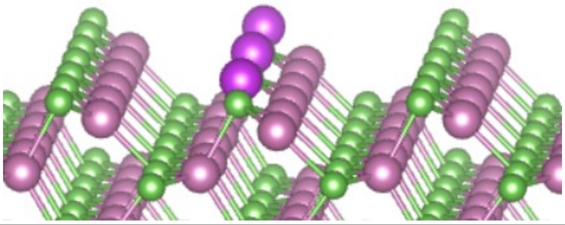 |
| **In vacancy** | |
| 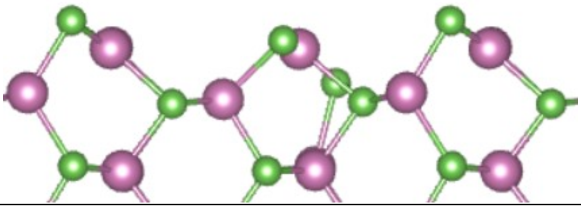 | 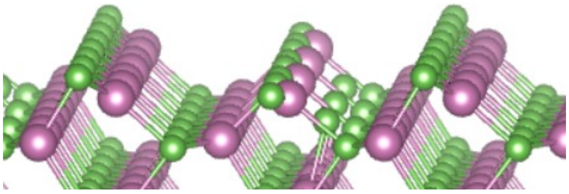 |
| **Bi substitutes In atom** | |
| 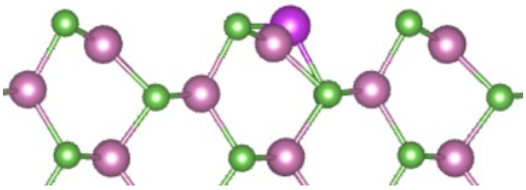 | 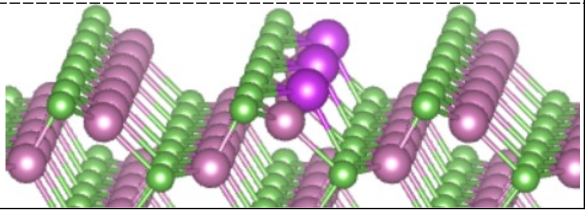 |

**References**

1 O. E. Tereshchenko, D. Paget, A. C. H. Rowe, V. L. Berkovits, P. Chiaradia, B. P. Doyle and S. Nannarone, *Surf. Sci.*, 2009, **603**, 518–522.

2 K. Szamota Leandersson, M. Göthelid, O. Tjernberg and U. O. Karlsson, *Appl. Surf. Sci.*, 2003, **212–213**, 589–594.

3 G. R. Bell, N. S. Kaijaks, R. J. Dixon and C. F. McConville, *Surf. Sci.*, 1998, **401**, 125–137.

4 V. De Renzi, M. G. Betti, V. Corradini, P. Fantini, V. Martinelli and C. Mariani, *J. Phys.*

*Condens. Matter*, 1999, **11**, 7447–7461.

5 M. G. Betti, D. Berselli and C. Mariani, *Phys. Rev. B*, 1999, **59**, 760–765.

6 A. Höglund, C. W. M. Castleton, M. Göthelid, B. Johansson and S. Mirbt, *Phys. Rev. B - Condens. Matter Mater. Phys.*, 2006, **74**, 1–10.

7 D. Y. Zemlyanov, M. Jespersen, D. N. Zakharov, J. Hu, R. Paul, A. Kumar, S. Pacley, N. Glavin, D. Saenz, K. C. Smith, T. S. Fisher and A. A. Voevodin, *Nanotechnology*, , DOI:10.1088/1361-6528/aaa6ef.
